# Investigating Dual-Species *Candida auris* and Staphylococcal Biofilm Antiseptic Challenge

**DOI:** 10.3390/antibiotics11070931

**Published:** 2022-07-11

**Authors:** Dolunay Gülmez, Jason L. Brown, Mark C. Butcher, Christopher Delaney, Ryan Kean, Gordon Ramage, Bryn Short

**Affiliations:** 1 Medical Microbiology Department, Faculty of Medicine, Hacettepe University, Ankara 06100, Turkey; 2 School of Medicine, Dentistry and Nursing, College of Medical, Veterinary and Life Sciences, Glasgow Dental Hospital, Glasgow University, Glasgow G2 3JZ, UK; jason.brown@glasgow.ac.uk (J.L.B.); 2135158B@student.gla.ac.uk (M.C.B.); christopher.delaney@glasgow.ac.uk (C.D.); gordon.ramage@glasgow.ac.uk (G.R.); 3 Department of Biological and Biomedical Sciences, School of Health and Life Sciences, Glasgow Caledonian University, Glasgow G2 3JZ, UK; ryan.kean@gcu.ac.uk

**Keywords:** biofilm, polymicrobial, *Candida auris*, antiseptic, *Staphylococcus* spp.

## Abstract

*Candida auris* can persistently colonize human skin, alongside a diverse bacterial microbiome. In this study we aimed to investigate the efficacy of antiseptic activities on dual-species interkingdom biofilms containing staphylococci to determine if antiseptic tolerance was negatively impacted by dual-species biofilms. Chlorhexidine, povidone iodine, and hydrogen peroxide (H_2_O_2_), were able to significantly reduce biofilm viable cell counts following exposure at 2%, 10%, and 3%, respectively. Notably, H_2_O_2_-treated biofilms were able to significantly recover and considerably repopulate following treatment. Fortunately, inter-kingdom interactions in dual-species biofilms of *C. auris* and staphylococci did not increase the tolerance of *C. auris* against antiseptics in vitro. These data indicate mixed infections are manageable with chlorhexidine and povidone iodine, but caution should be exercised in the consideration of H_2_O_2_.

## 1. Introduction

In the decade since its spontaneous emergence, *Candida auris* has quickly established itself as a prolific healthcare pathogen. With outbreaks recorded in six continents, *C. auris* has spread rapidly throughout healthcare environments, displaying worrying characteristics such as multi-drug resistance and the ability to survive on surfaces for at least 2 weeks [1]. Our inability to diagnose this pathogen accurately and consistently contributes to its continued spread within healthcare settings. Within healthcare environments, live *C. auris* cells have been recovered from the surrounding area of colonised patients such as mattresses, bedside tables, and chairs [2]. There is growing evidence corroborating these findings that also reveal the ability of *C. auris* to tolerate clinically relevant concentrations of antiseptics such as chlorhexidine (CHX) and hydrogen peroxide (H_2_O_2_) [3].

*C. auris* most commonly colonises the skin of both healthy and immunocompromised individuals, and therefore inevitably encounters members of the skin microflora [4]. *Staphylococcus* has been described as the dominant genus of the skin microflora with increases in the abundance of species such as *S. aureus* being observed in wound infections [5]. Other pathogenic fungi such as *C. albicans* have extensively shown their ability to interact with bacteria that comprise part of the skin microbiota [6]. These interactions are commonplace within biofilms and often result in increases of virulence factors, such as adhesins or drug resistance mechanisms [6]. Previous studies have also shown *C. auris* isolates are capable of forming robust biofilms where cell phenotype significantly influences virulence and biofilm quality [7].

There is a significant lack of information on whether *C. auris* interacts with other fungi or bacteria despite it occupying the same niche as other fungi and bacteria. It has been shown that *C. albicans’* extracellular matrix is capable of coating and protecting *S. aureus* from the effects of vancomycin treatment [8], so we hypothesised that dual-species interactions with *C. auris* and *S. aureus* may mirror this effect and could have implications for skin and wound infections. Therefore, we herein aimed to investigate whether the interaction between *C. auris* and either *S. aureus* or *S. epidermidis* within a dual-species biofilm model influences the tolerance profile of *C. auris* to the commonly used antiseptics CHX, H_2_O_2_, and povidone iodine (PVP-I).

## 2. Results

**Figure 1:**
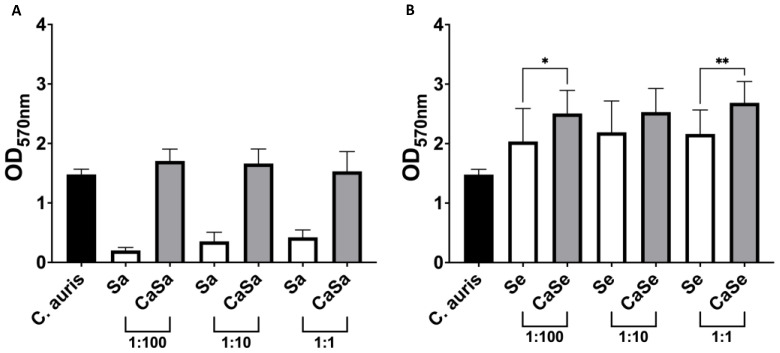
***Staphylococcus aureus* does not influence biomass of dual-species biofilms.** Biofilms were grown in growth media at varying ratios of bacteria to fungi. The concentration of *C. auris* (Ca) remained constant at 1 × 10^6^ cells/mL whereas *S. aureus* (Sa; **A**) and *S. epidermidis* (Se; **B**) were added to growth media at final concentrations of 1 × 10^4^, 1 × 10^5^ and1 × 10^6^ cells/mL. Ratios provided below the *x*-axis indicate the number of bacterial to fungal cells. Biofilms were washed with PBS and stained with crystal violet before measuring the absorbance of bound dye at 570 nm. Experiments were performed in triplicate and error bars represent the standard deviation of the mean (*; *p* < 0.05 **; *p* < 0.01).

**Figure 2:**
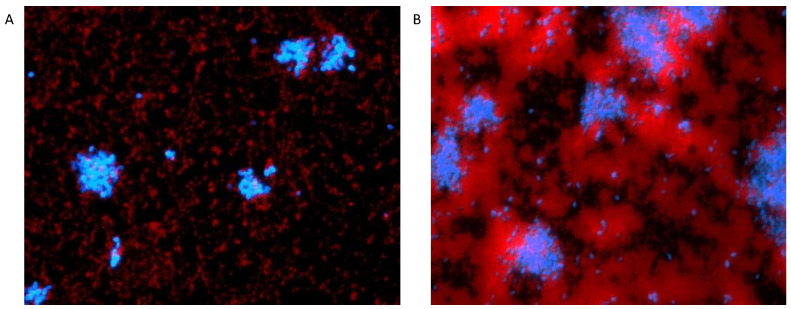
**Development of a *Candida auris* dual-species biofilm.** Images captured of 24 h dual-species biofilms containing *C. auris* with *S. aureus* (**A**) and *S. epidermidis* (**B**). Biofilms were stained using 1.5 mM calcofluor white and 1.5 mM hexidium iodide for 1 h to visualise fungi (blue) and bacteria (red). Calcofluor white and hexidium iodide were fluoresced at excitation/emission wavelengths of 357/447 and 531/593 nm, respectively.

Bacteria and fungi are commonly isolated together in healthcare environments, and the interactions that take place within these biofilms can drive antimicrobial resistance [6]. However, interactions between *C. auris* and bacteria are yet to be explored. Therefore, a dual-species biofilm model containing *C. auris* and the prolific skin pathogens *S. aureus* and *S. epidermidis* was developed. Multiple ratios of bacteria to fungi were assessed to ensure that bacteria did not inhibit fungal growth. It was observed that shifting ratios of *S. aureus* did not significantly alter biofilm growth and biomass (Figure 1A). Interestingly, dual-species biofilms grown with *C. auris* and *S. epidermidis* showed a 1.2-fold increase in biomass compared to *S. epidermidis* alone when bacterial cells were 100-fold fewer and where both *C. auris* and *S. epidermidis* were present in equal numbers (Figure 1B). As shifting ratios of bacteria to fungi did not significantly alter the biofilm biomass of dual-species biofilms, a ratio of 1:1 at 1 × 10^6^ cells/mL was selected, which has been used previously in a dual-species biofilm between *Candida albicans* and *S. aureus* [6]. Dual-species biofilms at this inoculum concentration supported the growth of both bacteria and fungi, which is visually represented in Figure 2. From these data it is evident that *Staphylococcus* spp. do not inhibit *C. auris* growth.

**Figure 3:**
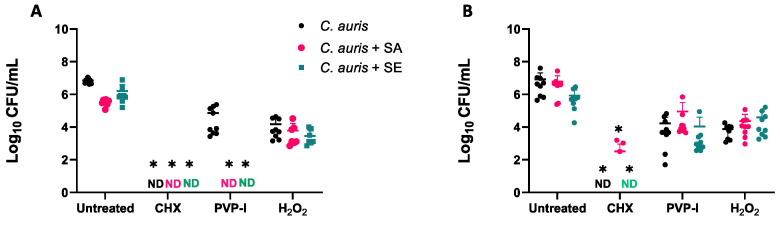

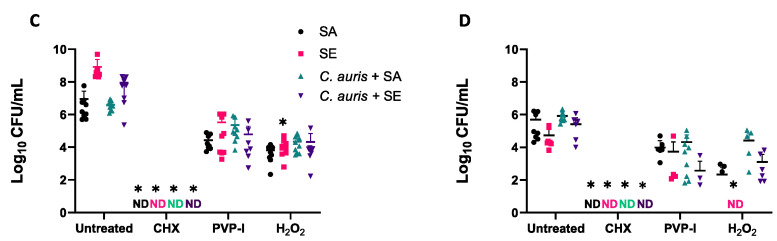
**Multi-species biofilms do not provide additional protection against antiseptics.** Single and dual-species biofilms of *C. auris* and either *S. aureus* (SA) or *S. epidermidis* (SE) were grown for 24 h before being challenged with 2% (*v/v*) CHX, 10% (*w/v*) PVP-I, or 3% (*v/v*) H_2_O_2_ for 5min with subsequent neutralisation with 5% (*v/v*) sodium thiosulphate. Immediately following neutralisation, biofilm cells were removed from their substrate, serially diluted, and plated onto SAB agar with erythromycin and ampicillin or LB agar with amphotericin B to select for fungi and bacteria, respectively. Viable *C. auris* cells were quantified after treatment on coverslips and cellulose matrix (**A** and **B**, respectively). Staphylococcal species were quantified following growth and treatment on coverslips and cellulose matrix (**C** and **D**, respectively). Experiments were repeated three times on separate occasions (*; *p* < 0.05). * denotes statistical significance when compared to the untreated control of the same biofilm.

As single and dual-species biofilms of both bacteria and fungi are typically associated with an increased tolerance to antimicrobial challenges, we next sought to test the ability of such biofilms to tolerate antiseptics. In all cases, the mean number of fungi and bacteria in each biofilm was reduced by at least 2 × log_10_ CFU/mL following treatment (Figure 3). CHX displayed the highest efficiency by significantly reducing viable cells of both *S. aureus, S. epidermidis,* as well as *C. auris* by more than 4 × log_10_ (*p* < 0.05). H_2_O_2_ was the least effective against fungi within single and multi-species biofilm environments. H_2_O_2_ prevented the growth of *S. epidermidis* biofilms when grown on cellulose matrices (Figure 3D), but all other biofilms survived treatment with more than 1 × 10^2^ CFU/mL remaining within the biofilm. Treatments with 10% PVP-I were able to reduce biofilm viability and also showed a greater efficacy against *C. auris* biofilms grown on plastic coverslips compared to cellulose matrices (Figure 3A; *p* < 0.05). 

**Figure 4:**
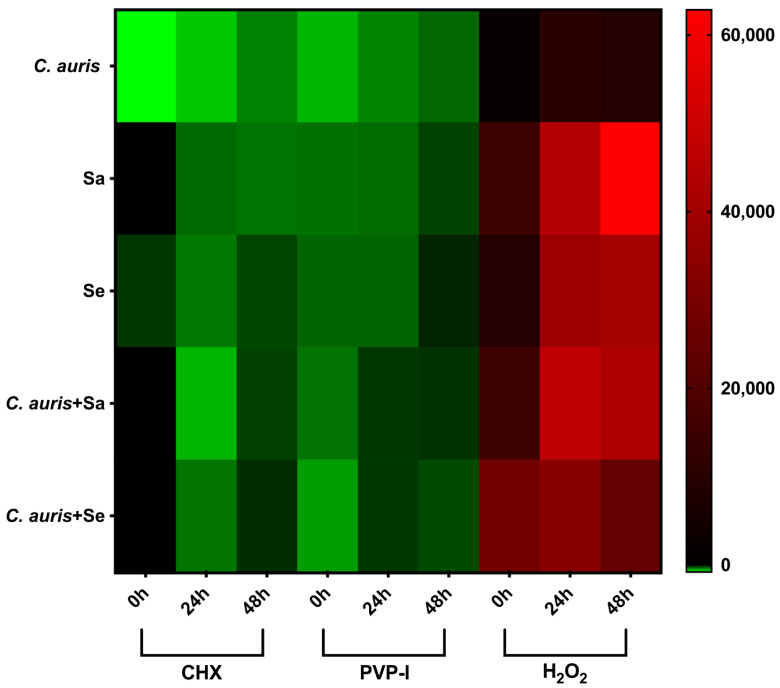
**Hydrogen peroxide is ineffective at preventing biofilm regrowth.** Mature *C. auris* biofilms +/− *S. aureus* (SA) and *S. epidermidis* (SE) were treated with 2% (*v/v*) CHX, 10% (*w/v*) PVP-I, or 3% (*v/v*) H_2_O_2_ for 5 min before a 15 min neutralisation step using 5% (*w/v*) sodium thiosulphate. Immediately after neutralisation, biofilm viability was assessed using 0.01% (*w/v*) resazurin sodium salt diluted in growth media. Additional biofilms were included to monitor biofilm re-growth. Fresh growth media was added to each biofilm, which was then incubated for a further 24 and 48 h. Biofilm viability was measured at each time point using 0.01% (*w/v*) resazurin sodium salt. Experiments were performed in triplicate on three separate occasions.

As shown above, each antiseptic was able to reduce the total number of viable cells within each type of biofilm. Therefore, we then assessed their ability to tolerate and regrow following treatment (Figure 4). CHX and PVP-I were again confirmed as the most potent agents against both single and dual-species biofilms, with residual regrowth occurring over 48 h. After 48 h of regrowth, dual-species biofilms treated with 3% (*v/v*) H_2_O_2_ recovered considerably. Biofilms containing *C. auris* and *S. aureus,* or *C. auris* and *S. epidermidis*, were found to be 4.5- and 2.6-fold higher, respectively, than single species *C. auris* biofilms.

## 3. Discussion

Interactions between fungi and bacteria have been shown to enhance antimicrobial tolerance [6,8,9]. Due to this, polymicrobial infections are responsible for higher mortality rates than their mono-microbial counterparts [6]. Any interactions that may take place between *C. auris* and other fungi or bacteria are yet to be characterised. We therefore aimed to begin to identify whether *C. auris* interacts with staphylococci species within a biofilm and if these interactions influence the resistance profile of the biofilm. *C. auris* biofilms have recently been shown to tolerate antiseptic treatments at clinically relevant concentrations [3]. Levels of tolerance to antiseptics were dependant on biofilm complexity, with developing biofilms being more susceptible to CHX and H_2_O_2._ However, *C. auris* was still recovered from the skin of hospitalised patients receiving daily CHX washes [4]. This decreased susceptibility to CHX in clinical settings may be a result of protective interactions with other non-staphylococcal members of the skin microbiome. In 2017, Moore and colleagues showed that *C. auris* was more susceptible to 2% (*v/v*) CHX with 70% (*v/v*) isopropyl alcohol [10]. Despite these promising results, the fungicidal activity of this antiseptic–alcohol mixture was observed in cells in suspension. Therefore, the activity of this treatment on single and multi-species *C. auris* biofilms is unknown. 

In this study *C. auris* was shown to be less responsive to PVP-I compared to previous studies [3,10]. As discussed above, Moore and colleagues tested the *C. auris* response to antiseptic challenge in planktonic cells, which may explain their increased susceptibility [10]. Furthermore, our group reported PVP-I to be highly effective against mature *C. auris* biofilms, though this was from a different strain [3]. Indeed, we have previously shown that biofilms formed by *C. auris* NCPF 8973 (the strain used in this study) formed a more robust biofilm than all other strains tested [7]. Levels of glucans and mannans within the *C. auris* biofilm matrix have recently been characterised and differences between strains were observed [11]. Taken together, strain-specific biofilm heterogeneity can ultimately lead to varying responses of *C. auris* to antiseptics such as PVP-I.

Herein, we demonstrated a reduced activity of H_2_O_2_ against single and dual-species biofilms and similar findings have been reported elsewhere [3]. *C. auris* has been reported to contain catalase genes while both *S. aureus* and *S. epidermidis* display catalase activity that can be utilized as a survival factor against osmotic stressors and a H_2_O_2_ challenge, indicating why H_2_O_2_ is less effective than CHX and PVP-I [12,13,14].

Although there is no observed increase in biofilm tolerance, growth within a dual-species biofilm appeared to drive *C. auris* to form small aggregates. The ability of cells to form aggregates has been characterised in cystic fibrosis and chronic wounds [15]. Although not directly adhered to surfaces, these cells display biofilm traits such as recalcitrance to antimicrobial therapies [16]. Therefore, existing within a dual-species environment may increase the ability of non-aggregating *C. auris* to tolerate disinfection protocols and drive spread throughout healthcare environments. 

This small study has only used a single *C. auris* strain, which is a member of the Indian clade. To fully characterise levels of antiseptic tolerance between *C. auris* and staphylococci, additional *C. auris* strains should be used. We also did not include a physical challenge in addition to the chemical action of the antiseptics used (e.g., wiping), which would likely affect cell survival rates. The data discussed herein reveal that interactions between non-aggregating *C. auris* and *Staphylococcus* species do not increase biofilm biomass, which is reassuring for the management of these infections. Notably however, the observed increase in cellular regrowth within the H_2_O_2_ treated dual-species biofilm indicates that bacteria have the potential to influence the ability of *C. auris* to persist within healthcare environments. Further research is required to fully understand the intercellular interactions that take place between *C. auris* and other bacterial species.

## 4. Conclusions

Being frequently isolated from the skin of both healthy and immunocompromised patients, the opportunities for *C. auris* to interact with bacteria that also occupy the same niche. Data herein show that *Staphylococcus* spp. do not promote a more tolerant phenotype in *C. auris.* However, similar interactions between staphylococci and *C. albicans* increase both fungal and bacterial virulence. Therefore, further studies may reveal other effects of these interactions.

## 5. Materials and Methods

### 5.1. Microbial Growth and Standardisation

*C. auris* isolate NCPF 8973 (Indian clade), *S. aureus* NCTC 10,833, and *S. epidermidis* RP62A (ATCC 35984) were used throughout this study. *C. auris* was grown on Sabouraud’s Dextrose (SAB) agar (Sigma-Aldrich, Dorset, UK) at 30 °C for 48 h and bacterial strains were grown for 24 h at 37 °C on Luria Bertoni (LB) agar (Sigma-Aldrich). All microorganisms on agar were stored at 4 °C. Yeast and bacterial colonies were suspended in yeast peptone dextrose media (YPD; Sigma-Aldrich) or LB broth, respectively. Broths were incubated for 18 h overnight before washing twice with phosphate-buffered saline (PBS; Sigma-Aldrich). Staphylococcal cells were diluted to 0.6 OD_600_ (approx. 1 × 10^8^ cells/mL) and yeast cells were counted using a haemocytometer. Microorganisms were diluted to assay specific concentrations in desired media.

### 5.2. Biofilm Growth, Quantification, and Visualisation

Fungal and bacterial broths were standardised as described above. *C. auris* was adjusted to 1 × 10^6^ cells/mL as done so previously [7,17]. Bacteria were diluted to concentrations ranging from 1 × 10^4^ to 1 × 10^6^ CFU/mL. Cell suspensions were prepared as single or dual species inoculums in Todd Hewitt broth (THB; Thermo Fisher, Paisley, UK) supplemented with hemin and menadione, mixed 1:1 with Roswell Parks Memorial Institute (RPMI) media (Sigma-Aldrich). Biofilms were grown in treated 96-well, flat-bottom, microtitre plates (Thermo Fisher) and incubated at 37 °C for 24 h. Biofilm formation was assessed via crystal violet staining as described previously [6].

Bioflims were visualised by fluorescently labelling microorganisms with 1.5 mM hexidium iodide (Fisher Scientific, Loughborough, UK) and 1.5 mM calcofluor white (Thermo Fisher). Biofilms were incubated with the fluorescent dyes at 37 °C for 1 h. Biofilms were washed twice with PBS to remove excess dye and imaged using an EVOS cell imaging system (Thermo Fisher) at 40× magnification. Calcofluor white and hexidium iodide fluorescence was detected at excitation/emission wavelengths of 357/447 and 531/593 nm, respectively, before overlaying the images.

### 5.3. Hydrogel Preparation and Biofilm Growth on Cellulose Matrix

A 3-dimensional biofilm model was used as described previously by our group [3]. Briefly, this was prepared using 10% 3-sulfopropyl acrylate potassium salt, 0.95% poly(ethylene glycol) deacrylate, 0.01% 1-hydroxycyclohexyl phenyl ketone, and 50% horse serum diluted using 2× PBS pH 7.3. The mixture was added to treated, flat-bottomed, 12-well plates (Thermo Fisher) and gels were polymerised under 365 nm ultraviolet light for 30 min. For biofilm growth, microorganisms were standardised to 1 × 10^7^ cells/mL in PBS and incubated for 2 h with the required number of cellulose matrix pieces (cut to 1.25 cm^2^) at 37 °C with agitation. Sections of matrix were placed on top of the gels and incubated for a further 24 h at 37 °C.

### 5.4. Biofilm Treatment and Re-Growth Assessment

Single and multi-species biofilms on coverslips and cellulose matrices were exposed to 2% (*v/v*) CHX, 10% (*w/v*) PVP-I, or 3% (*v/v*) H_2_O_2_ for 5 min, and neutralised using 5% (*w/v*) sodium thiosulphate as described previously [3]. Biofilm viability was quantified directly after treatment using 0.01% (*w/v*) resazurin sodium salt using the method outlined above, and by colony forming unit (CFU) quantification. Biofilm biomass was removed from the growth substrate via sonication in an ultrasonic water bath (Fisher Scientific, Loughborough, UK) for 10 min at 35 kHz. The biomass was subsequently serially diluted 10-fold in an untreated, round-bottom microtitre plate as described elsewhere [17] and plated onto selective agars. *C. auris* was selected for using SAB agar supplemented with 8 µg/mL amoxicillin and 75 µg/mL erythromycin, and LB agar supplemented with 64 µg/mL amphotericin B was used to select for *S. aureus* and *S. epidermidis* growth [18].

To assess biofilm re-growth, fresh growth media was added back to the treated biofilms and then incubated for a further 48 h. Every 24 h biofilm viability was quantified by diluting a 1% (*w/v*) stock of resazurin sodium salt 1:100 in biofilm growth media. Diluted resazurin salt was incubated with biofilms for 1 h. Media fluorescence was measured at an excitation/emission of 530/590 nm in a microtitre plate reader (FLUOStar Omega, BGM Labtech, Aylesbury, UK). 

### 5.5. Statistical Analysis

All experiments were carried out in triplicate on three independent occasions. Positive untreated controls were included for comparison to treated biofilms, and negative controls were used to test for contamination. Statistical analysis and graph production was performed using GraphPad Prism (Version 8; La Jolla, CA, USA) and SPSS Statistics (Version 26.0; IBM, Chicago, IL, USA). Kruskal–Wallis tests were used to compare means of biofilm metabolic activity and biofilm CFUs before and after treatment. Differences between means were deemed significant where *p* < 0.05.

## Data Availability

The data presented in this study are available on request from the corresponding authors.
